# Benefits of Dual-Layer Spectral CT Imaging in Staging and Preoperative Evaluation of Pancreatic Ductal Adenocarcinoma

**DOI:** 10.3390/jcm12196145

**Published:** 2023-09-23

**Authors:** Constantin Ehrengut, Timm Denecke, Hans-Jonas Meyer

**Affiliations:** Klinik und Poliklinik für Diagnostische und Interventionelle Radiologie, Universitätsklinikum Leipzig, 04103 Leipzig, Germany; constantin.ehrengut@medizin.uni-leipzig.de (C.E.);

**Keywords:** PDAC, spectral CT, dual layer, dual energy

## Abstract

Imaging of pancreatic malignancies is challenging but has a major impact on the patients therapeutic approach and outcome. In particular with pancreatic ductal adenocarcinoma (PDAC), usually a hypovascularized tumor, conventional CT imaging can be prone to errors in determining tumor extent and presence of metastatic disease. Dual-layer spectral detector CT (SDCT) is an emerging technique for acquiring spectral information without the need for prospective patient selection or specific protocols, with a detector capable of differentiating high- and low-energy photons to acquire full spectral images. In this review, we present the diagnostic benefits and capabilities of modern SDCT imaging with a focus on PDAC. We highlight the most useful virtual reconstructions in oncologic imaging and their benefits in staging and assessment of resectability in PDAC, including the assessment of tumor extent, vascular infiltration, and metastatic disease. We present imaging examples on a latest-generation SDCT scanner.

## 1. Introduction

Pancreatic ductal adenocarcinoma (PDAC) is a prevalent malignant tumor with the seventh-largest cancer-related mortality worldwide [1]. Five-year overall survival is still poor, with only 4% in 2011 according to a recent SEER database analysis [2]. The only curative treatment option is complete surgical removal of the tumor. However, most patients present with more advanced, unresectable stages at the initial time of diagnosis, with only palliative treatment options left. Computed tomography (CT) and magnetic resonance imaging (MRI) play a crucial role in evaluating disease extent and identifying patients suitable for surgical resection. CT is highly sensitive for detecting PDAC; however, studies suggest a considerable amount of visually iso-attenuating tumors that can easily be missed, especially in CT protocols without a pancreatic parenchymal contrast phase [3]. PET-CT has also been reported to be highly sensitive for the detection of PDAC; the specificity, however, is relatively low and varies greatly among studies, possibly due to the confusion with inflammatory disease such as mass-forming pancreatitis [4]. Recent studies have shown that dual-energy CT improves the detection of tumors, has greater diagnostic accuracy in determining tumor extent and vascular involvement and increases sensitivity and specificity in detecting liver metastases [5,6,7,8]. Dual-layer spectral detector CT (SDCT) is an emerging technique utilizing a detector capable of differentiating low- and high-energy photons and acquiring spectral information without the need for additional X-ray tubes or specific protocols. In this review, the benefits of SDCT and the most useful virtual reconstructions in oncologic imaging are discussed and its benefits in staging and preoperative evaluation of PDAC with imaging examples on a latest-generation SDCT scanner are shown.

## 2. Dual-Energy Techniques

With spectral imaging, non-invasive information about the composition of specific materials is possible. This allows for selective quantification of elements such as iodine and calcium. To achieve this, the attenuation of at least two different predefined X-ray beam spectra must be acquired (dual-energy scan) instead of one whole integrated beam spectrum attenuation as used in conventional CT imaging. For spectral CT, this can be realized in several construction options of the scanner system, regarding either the X-ray source or the detector design. On the source side, dual-energy CT systems use two X-ray tubes with different tube currents (dual-source systems), a single X-ray tube with kV switching, or a single tube with an additional filter to split the beam in two distinct spectra (dual-beam/twin beam) [9]. The newest technique in acquiring spectral information is Photon-counting detector CT (PDCT). This technique utilizes a direct conversion X-ray detector, where incoming X-ray photon energies are directly and separately converted into electronic signals. This allows for improvements in spatial resolution and radiation dose reduction, while still generating full spectral images. However, PDCT is an emerging technique with very limited availability and currently only one commercially available system.

### Detector Based Dual-Energy Systems (SDCT)

SDCT offers a solution where only one X-ray tube is necessary to generate full-spectrum images. It utilizes a detector consisting of two layers. The upper layer absorbs low-energy photons and is permeable for high-energy photons, while the layer beneath is sensitive for the remnant high-energy photons. This allows for the calculation of full-spectrum images in every examination (always on) with excellent spatial and temporal resolution, while eliminating the need for additional tubes or specific protocols and parameter settings [10,11]. Acquisition can be performed with full field of view and rotation speed and there are no restrictions regarding radiation dose-reduction strategies as available in conventional CT. Downsides include longer reconstructions times and lower spectral contrast compared to dual-source systems. The conventional images are generated through filtered back projections or iterative reconstruction algorithms. The image quality of these conventional images is comparable to images obtained from a single-energy scanner. A phantom study comparing SDCT to conventional CT showed higher qualitative scores of enhancement, noise, and image quality for SDCT compared to a single-energy CT scanner. Noise ratios were also better in most anatomical regions [12]. The possibility to acquire spectral information in every scan without the need for prospective patient selection is a major benefit of SDCT and could benefit patients where the indications of the referral did not suggest the use of spectral CT in the first place. PDAC symptoms are rather unspecific and patients often undergo single-phase protocols without the apparent need for spectral information.

## 3. Virtual Reconstructions

After the scan, spectral information is used to calculate various digital reconstructions. The most investigated reconstructions so far are iodine density maps (IDM), virtual non-contrast imaging (VnC), virtual non-calcium imaging (VnCa) and virtual monoenergetic imaging (VMI). Of these VMI, IDM and VnC were shown to have the greatest diagnostic benefits in oncologic imaging [13]. Table 1 gives a brief summary of available virtual reconstructions and their benefits for oncologic imaging.

### 3.1. Virtual Monochromatic Images (VMI)

In virtual monochromatic imaging, post-processing algorithms create a virtual monochromatic X-ray beam usually in a range from 40 to 200 keV. With lower virtual monochromatic beam energies, the X-ray absorption of specific materials such as iodine is amplified by approaching the elements k-edge. The k-edge defines a specific X-ray energy in which a sudden increase in the X-ray absorption of a specific material is observed. The k-edge of iodine is 33.2 keV, thus lowering the virtual beam energy to values close to those that enhance iodine visualization and can provide better lesion-contrast ratios. This effect was shown to be useful in multiple oncologic settings. It helps in detecting and differentiating liver lesions [16,17,18] and facilitates washout characterization in hypervascularized liver lesions for the detection of HCC [19]. In hypervascularized renal lesions, altering the monoenergetic beam energy has a direct impact on the diagnostic accuracy, with a reduction in detectability in VMI energies above 70 keV [14]. As mentioned above, in PDAC, multiple studies suggested improvements in determining tumor extent and vascular involvement and increased sensitivity and specificity in detecting liver metastases. VMI has its limitations as image noise is increased at lower keV settings. However, noise reduction algorithms in dual-layer spectral CT can alleviate that effect and, in phantom studies, even showed an improved signal-to-noise ratio and better subjective image quality in simulated obese patients than polychromatic images, even at low energy levels (40 to 75 keV) [30]. High-energy VMI images also have their use as energies above ~110 keV reduce artifacts caused by metal implants, which can further be improved by the implementation of artifact reduction algorithms [31,32,33].

### 3.2. Iodine Density Maps (IDM)

With the addition of material decomposition algorithms, subtraction or highlighting of specific elements such as iodine or calcium is possible. With iodine being the major component of contrast media, iodine-based spectral reconstructions allow for direct visualization (or subtraction) of vascularization and perfusion in contrast-enhanced CT images [34]. Multiple studies have explored the usefulness of adding iodine density maps in oncologic imaging. Similar to VMI, IDM can help differentiate hypo- or hypervascularized lesions from surrounding parenchyma by highlighting contrast enrichment (or the lack of it) [20]. There are also other interesting applications for iodine-based material decomposition maps. A study from Drljevic-Nielsen et al. showed that intralesional iodine concentration was a significant predictor of a better treatment response and improved survival in renal cell carcinomas [21]. In gastric adenocarcinoma, iodine concentration was a beneficial prognostic marker as it correlated with angiogenesis and degree of differentiation [35]. Post-treatment spectral CT with iodine maps can help with monitoring treatment response after therapy as a possible surrogate marker for tumor vitality [22,23].

### 3.3. Virtual Non-Contrast Imaging (VnC)

With the above-mentioned material decomposition algorithms, virtual non-enhanced images can be generated by subtracting iodine from the enhanced CT image. The VNC image can replace true unenhanced images to reduce radiation or supplement an enhanced image protocol in case of incidental findings, where an unenhanced image is needed for further interpretation. It has shown equal sensitivity to true enhanced images in the detection of active hemorrhage [36] and could reliably replace true unenhanced images in CT cholangiography and in detection of urolithiasis [37,38]. Oncologic applications include helping to differentiate adrenal malignancies from adenomas (when combined with IDM) [25], evaluating treatment response after chemotherapy by helping to differentiate therapy induced calcifications from intralesional hemorrhage [26] and quantify post-ablation defects after radio frequency ablation [27]. It should be noted, however, that multiple studies suggest a significant difference in HU values of true and virtual unenhanced images, especially in abdominal imaging in regions with high fat, iodine or calcium content [39,40,41].

### 3.4. Virtual Non-Calcium Imaging (VnCa)

Using material decomposition algorithms to virtually subtract calcium has extensively been studied in orthopedic applications for the early detection of bone marrow edema in suspected trauma [42,43,44]. In conventional CT, the bone marrow is masked by the high calcium content of the bone matrix, making it nearly impossible to detect bone marrow edema or differentiate osteoporotic lesions from malignant bone infiltration. In multiple myeloma, VnCa allowed better and earlier detection rates for myeloic bone lesions, possibly accelerating therapy induction [28,29].

## 4. Assessing Disease Extent and Resectability of PDAC with Spectral CT

### 4.1. Tumor Detection and Tumor Extent

In most institutions, conventional CT is used as the standard preoperative imaging technique in patients with suspected pancreatic cancer. It allows for fast and comprehensive assessment of the disease extent and plays an important role in determining resectability.

Usually, PDAC is a hypovascularized tumor, which can be detected as a hypoattenuating mass on multiphasic conventional CT. The current diagnostic algorithm consists of a multiphasic CT approach: The differentiation between the adjacent pancreatic tissue and the tumor, as well as peripancreatic arterial involvement can be best provided in a pancreatic parenchymal contrast phase (late arterial phase), whereas venous involvement and metastases are characterized in the portal venous phase. The parenchymal phase allows the best differentiation; however, differences in contrast can sometimes be very subtle and tumor margins may appear ill defined on conventional CT images. Isoattenuating tumors are quite common with PDAC, with a prevalence of approximately 11% [3] and they are associated with better survival rates after resection compared to hypovascularized lesions. Therefore, their early detection is crucial and can have a major impact on the patients outcome. Monoenergetic image reconstruction can improve discrimination of tumor margins by improving lesion contrast. Multiple studies recommended VMI energy levels around 40 keV to achieve the best contrast without a noticeable increase in noise levels. Nagayama et al. and Han et al. showed improved objective und subjective tumor delineation between 40 and 55 keV and a superiority of VMI in the portal venous phase over the conventional polychromatic parenchymal phase [5,15]. This is especially useful in clinical routine as PDAC symptoms are rather unspecific and patients often undergo single-phase protocols for evaluation of abdominal pathologies with the resulting lack of the parenchymal phase in those cases.

Iodine density maps were identified to be also useful for clearer differentiating hypo- or hyper-vascularized lesions and may add supplemental information for the assessment of devascularization as a surrogate parameter for treatment response following chemotherapy as mentioned above [23,24].

**Figure 1:**
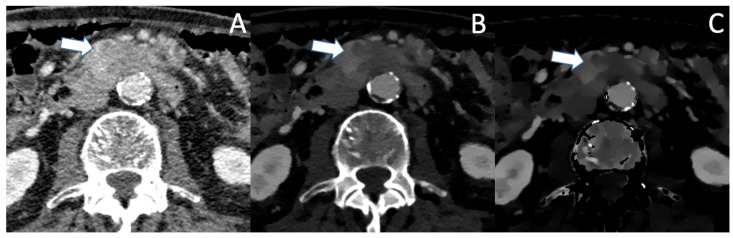
(**A**) The conventional CT image shows an ill-defined, moderately hypoattenuating mass of the pancreatic corpus in the portal venous phase (marked with the arrow). In VMI (**B**) and iodine density maps (**C**), the hypoattenuating tumor is more clearly visible with sharp demarcation to the adjacent pancreatic parenchyma.

A patient presenting with abdominal pain who received a single-phase protocol is shown in Figure 1, in which VMI and ID show a more clearly visible and well-demarcated lesion of the pancreatic corpus, which was later confirmed to be PDAC. Currently there are no studies directly comparing the benefits of SDCT to MRI in the detection of small or hypoattenuating lesions. However, several studies indicate good diagnostic performance of MRI in lesions <2 cm, with sensitivities ranging from 90 to 100% [45,46]. In particular, DWI has a good diagnostic performance with a sensitivity of 92–96% and specificity of 97–99% [47,48]; however, DWI has its limitations in differentiating mass-forming pancreatitis from PDAC, its similar ADC values [48]. With spectral CT, VMI and IDM can be used to improve the sensitivity and the specificity for the differentiation of mass-forming pancreatitis from PDAC [49].

**Figure 2:**
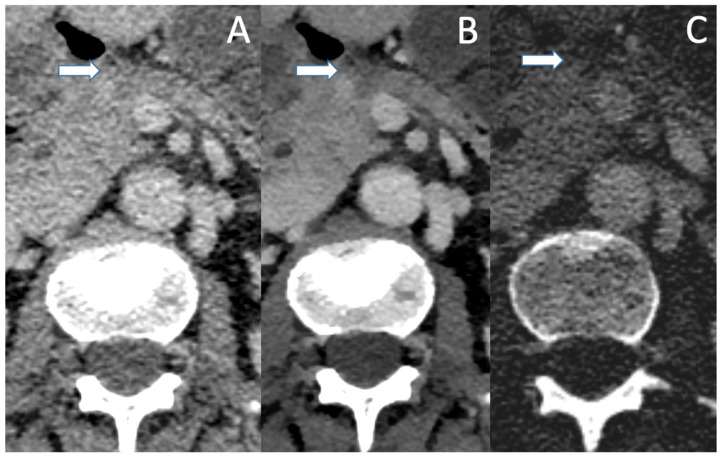
A patient with PDAC after neoadjuvant chemotherapy in conventional CT (**A**), VMI (**B**) and ID (**C**) in the portal venous phase. The small residual tumor (arrow) is barely visible in the conventional image and no statement about vascular association can be made. VMI and ID show a well-demarcated tumor which shows contact with the VMS, with no clear evidence of vascular infiltration.

In pretreated PDAC following neoadjuvant chemotherapy, efficacy of conventional CT in staging and evaluation of resectability is severely impaired, with studies showing underestimation of therapy response by overestimation of tumor size and vascular conflicts, possibly leading to falsely excluding patients from curative treatment [50]. More accurate delineation of tumor margins in a pretreated patient with PDAC in VMI and ID is shown in Figure 2. Spectral CT might especially be beneficial for those patients, though data on that matter are currently sparse and further studies are needed. Additionally, MRI with diffusion weighted imaging could provide a better differentiation between tumor tissue and fibrosis, but data on that matter currently only exist for other malignancies, such as rectal cancer [51].

### 4.2. Assessing Vascular Involvement

**Figure 3:**
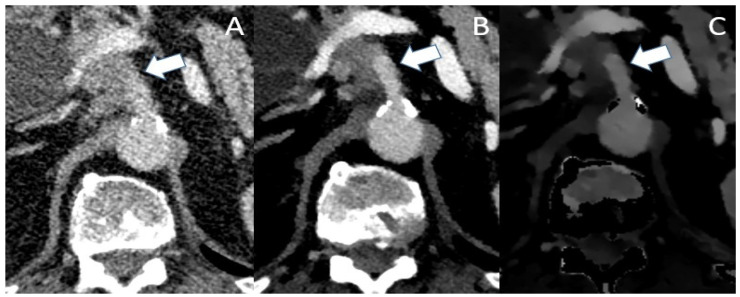
Conventional (**A**), VMI (**B**) and ID (**C**) of a patient with locally advanced PDAC. In the conventional image, infiltration of the celiac trunk (marked by the arrow) could be assumed with irregular opacifications within the vascular lumen. Though VMI and ID show the abutment of the celiac trunk, there is no clear evidence of intraluminal infiltration as the vascular lumen shows a sharp contrast with no opacifications.

**Figure 4:**
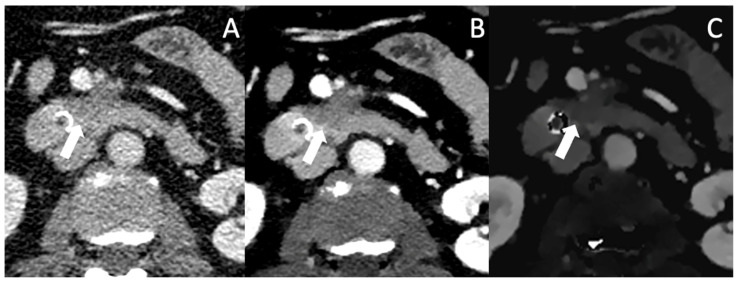
Conventional (**A**), VMI (**B**) and ID (**C**) of a patient with locally advanced PDAC (arrow). In VMI and ID, the lesion borders are well defined and the encasement of the AMS and the abutment of the VMS become more clearly visible.

One of the most important factors in determining resectability is vascular involvement. The exact interpretation of tumor contact with the celiac trunk, the superior mesenteric artery, the common hepatic artery as well as the superior mesenteric vein and the portal vein is crucial for assessing the different categories of resectability, as stated in the NCCN criteria [52]. Differentiation between contact, abutment (<180° contact) and encasement (>180° contact) of the vascular structures can be challenging in conventional CT, especially in pretreated patients as mentioned above. VMI helps with improving lesion contrast, tumor margins in association with the vascular structures appear sharper and subtle opacifications of the vascular lumen become visible (see Figure 3 and Figure 4). In the detection of vascular invasion, conventional CT has a sensitivity and specificity of 94% and 82.4%, with no statistically significant differences in the diagnostic performances of conventional pancreas protocol CT and contrast-enhanced MRI [45,53].

### 4.3. Detecting Metastases

To be eligible for curative treatment absence of distant metastatic disease is mandatory. The detection of small hypovascularized liver metastases can be challenging in conventional CT imaging. A recent meta-analysis of 987 patients showed an overall pooled sensitivity for CT of 45% for detection of liver metastases [54]. Though with a high risk of bias, the results suggest a severe disadvantage compared to MRI, which is still not a part of the routine diagnostic work-up in many centers. There are different techniques in conventional CT to improve contrast, such as lowering the tube voltage or optimizing the contrast medium injection. As aforementioned, iodine attenuation is increased with approaching the iodine k-edge, VMI should also improve differentiation between hypo- or hyper-vascularized liver lesions to the healthy liver parenchyma. Another study by Nagayama et al. showed superiority of VMI over the portal venous phases in lesions <1 cm with greater visibility of metastases in energies approaching the k-edge with nearly constant noise levels abroad the spectrum from 70 to 40 keV [7].

**Figure 5:**
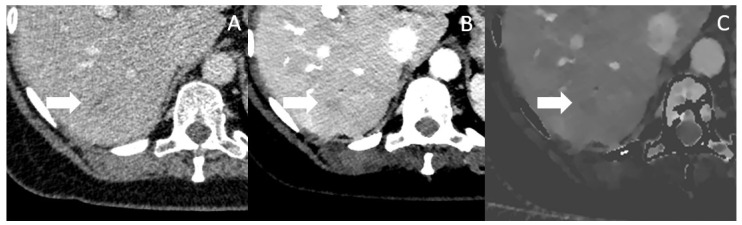
Conventional CT imaging with a subtle, ill-defined lesion in Segment VI (marked by the arrow) (**A**). In Mono-E (**B**) and on iodine density maps (**C**), the lesion is still subtle visible, but more clearly demarcated and hypoattenuating, increasing the likelihood for malignancy. The lesion was histopathologically a metastasis.

In Figure 5, a patient with a small hypodense lesion in Segment VI is depicted in conventional, VMI and IDM, which was later affirmed by biopsy to be a PDAC metastasis. Table 2 summarizes the above discussed benefits of spectral CT for the determination of tumor extent, vascular invasion, therapy monitoring and for the detection of metastasis and shows all available studies.

## 5. Conclusions

In this review, we highlighted the benefits of SDCT imaging in the diagnosis, staging and assessment of pancreatic ductal adenocarcinoma. SDCT is an emerging technique for acquiring full spectral information without the need for prospective patient selection or specific protocols. Several virtual reconstructions are useful in PDAC imaging. VMI in the portal venous phase can help differentiate pancreatic malignancies, vascular involvement, and liver metastases by increasing contrast differences and can be especially useful in protocols where the pancreatic parenchymal phase is missing. Iodine density maps can also improve visualization of hypo- or hypervascularized lesions and might aid in evaluation of treatment response. There is still a lack of, or scarce, evidence concerning the potential of SDCT in evaluation of vascular invasion, therapy monitoring and differentiation of PDAC mimics, such as mass-forming pancreatitis, but the few existing studies suggest potential benefits that need to be investigated further.

## Figures and Tables

**Table 1:** Summary of spectral reconstructions and their clinical applications.

Virtual Reconstructions	Technique	Clinical Applications	Benefits for Oncologic Imaging
Virtual Monochromatic Imaging (VMI)	Virtual monochromatic beam energies ranging from 40–200 keV	Artifact reduction Enhancing arterial contrast on venous scans Optimization of image quality Enhanced contrast ratios	Improved tumor delineation in hypo- or hypervascularized lesions [5,14,15] Improved detection of small metastasis [7] Differentiation of liver lesions [16,17,18,19]
Iodine Density Maps (IDM)	Material decomposition algorithm with quantitative iodine assessment and visualization	Enhanced contrast ratios Visualization of vascularization and perfusion	Improved tumor delineation in hypo- or hypervascularized lesions [20] Prediction of treatment response [21] Therapy monitoring [22,23,24]
Virtual Non-Contrast Imaging (VnC)	Material decomposition algorithm with iodine subtraction	Replacement of true non-contrast images in - Detection of active hemorrhage - Detection of renal/gallbladder stones - Characterization of incidental lesions - Vascular imaging (e.g., aortic endoleaks)	Differentiation of adrenal malignancies [25] Therapy monitoring [26,27]
Virtual Non-Calcium Imaging (VnCA)	Material decomposition algorithm with calcium subtraction	Bone edema detection and differentiation of bone structure	Earlier detection of malignant bone infiltration [28,29]

**Table 2:** Benefits of spectral CT in staging, preoperative evaluation and therapeutic monitoring of PDAC.

	Virtual Reconstructions	Studies	Results
Tumor detection and tumor extent	VMI/IDM	Nagayama Y et al. Eur Radiol. 2020 [15]	Patients: 48 Patients with PDAC. SDCT (Philips) Results: Image noise was significantly lower for VMI than for conventional CT (*p* < 0.01).CNR increased with decreasing energy, CNR, CNR were significantly greater for VMI than for conventional CT. Subjective image quality of VMI40–50 were equivalent/slightly better in portal venous phase than conventional pancreas parenchymal phase.
		Han et al. Journal of the Belgian Society of Radiology. 2022 [5]	Patients: 64 Patients with PDAC. SDCT (Philips) Results: VMI40 and VMI55 demonstrated higher tumor-to-pancreas CNR, attenuation difference, and higher peripancreatic vascular CNR and SNR than the pancreatic-phase image and VMI70 (*p* < 0.001). On subjective analysis, VMI55 showed the best tumor conspicuity
		Noda et al. Clinical Radiology 2020 [8]	Patients: 74 Patients. Dual Source (GE) Attenuation, background noise, SNR, and CNR peaked on VMIs at 40 keV (*p* < 0.0001) and gradually decreased with increasing energy levels. Measuring tumor sizes was better at VMIs at 40 keV and tended to be overestimated at higher energy levels. tumour conspicuity was also significantly superior on VMIs at 40 keV than at all other energy levels (*p* < 0.0001).
Therapy response	IDM	Kawamoto et al. Abdom Radiol (NY). 2018 [23]	Patients: 18 with PDAC. Dual source (Siemens) Results: Tumor iodine uptake and normalized tumor iodine uptake decreased in post-chemotherapy CT compared to the baseline (0.7 to 1.7 mg/mL at baseline and from 0.6 to 1.5 mg/mL at post-chemotherapy in arterial phase, and from 1.1 to 2.7 mg/mL at baseline and from 0.9 to 2.3 mg/mL at post-chemotherapy in venous phase.)
Detection of metastasis	VMI/IDM	Nagayama et al. Eur Radiol. 2019 [7]	Patients: 81 with hypovascular liver-metastases. SDCT (Philips) in portal venous Phase Results: - Image noise of VMI40–200 was consistently lower than that of conventional CT (*p* < 0.01). Tumor-to-liver contrast and CNR increased as the energy decreased with CNR at VMI40-70 which was higher than on PEI (*p* < 0.001) - Best subjective score for VMI40 and VMI50–70. - Lesion detectability at VMI40 was significantly superior to PEI, especially for lesions with diameters of <10 mm (*p* < 0.01, kappa ≥ 0.6).
Vascular infiltration	Currently no clinical data		
